# Therapeutic drug monitoring and TB treatment outcomes in patients with diabetes mellitus

**DOI:** 10.5588/ijtld.22.0448

**Published:** 2023-02-01

**Authors:** Y. Alkabab, J. Warkentin, J. Cummins, B. Katz, B. M. Denison, A. Bartok, A. Khalil, L. R. Young, E. Timme, C. A. Peloquin, D. Ashkin, E. R. Houpt, S. K. Heysell

**Affiliations:** 1 Medical University of South Carolina, Charleston, SC, USA; 2 Tennessee Department of Health, Nashville, TN, USA; 3 New Mexico Department of Health, Santa Fe, NM, USA; 4 Virginia Department of Health, Richmond, VA, USA; 5 Arizona Department of Health Services, Phoenix, AZ, USA; 6 University of Florida, Gainesville, FL, USA; 7 University of Virginia, Charlottesville, VA, USA

**Keywords:** TB, DM, treatment outcomes, sputum culture conversion, therapeutic drug monitoring

## Abstract

**BACKGROUND:**

Diabetes mellitus (DM) increases the risk of TB disease and poor treatment outcomes such as delayed sputum culture conversion due to inadequate drug exposure. Therapeutic drug monitoring (TDM) has improved these outcomes in some settings.

**METHODS:**

To compare treatment outcomes in programs with routine TDM vs. programs that did not use TDM, we conducted a retrospective study among people with DM and TB at health departments in four US states.

**RESULTS:**

A total of 170 patients were enrolled (73 patients in the non-TDM group and 97 patients in the TDM group). Days to sputum culture conversion and total treatment duration were significantly shorter in the TDM group vs. the non-TDM group. In adjusted analyses, patients who underwent TDM were significantly more likely to achieve sputum culture conversion at 2 months (*P* = 0.007).

**CONCLUSION:**

TDM hastened microbiological cure from TB among people with DM and a high risk for poor treatment outcomes in the programmatic setting.

## Abstract (fr)

**CONTEXTE:**

Le diabète sucré (DM) augmente le risque de TB et de mauvais résultats thérapeutiques, tels qu’un retard de conversion des cultures d’expectorations dû à une exposition inadéquate aux médicaments. Le suivi thérapeutique pharmacologique (TDM) a permis d’améliorer ces résultats dans certains contextes.

**MÉTHODES:**

Afin de comparer les résultats thérapeutiques dans les programmes ayant systématiquement recours au TDM et dans ceux n’ayant pas recours au TDM, nous avons réalisé une étude rétrospective parmi les patients atteints de DM et de TB dans les services de santé de quatre États américains.

**RÉSULTATS:**

Au total, 170 patients ont été inclus (73 patients dans le groupe sans TDM et 97 patients dans le groupe avec TDM). Le nombre de jours avant conversion des cultures d’expectorations et la durée totale du traitement étaient significativement plus courts dans le groupe avec TDM que dans le groupe sans TDM. Dans les analyses ajustées, les patients ayant bénéficié du TDM étaient significativement plus susceptibles d’obtenir une conversion des cultures d’expectorations à 2 mois (*P* = 0,007).

**CONCLUSION:**

Le TDM a accéléré la guérison microbiologique de la TB chez les personnes atteintes de DM et présentant un risque élevé de mauvais résultats thérapeutiques dans un contexte programmatique.

A curable and preventable infectious disease, TB remains one of the top 10 causes of death globally and is projected to worsen due to the COVID-19 pandemic.^1^ In the United States, the incidence of TB disease in 2021 was 2.4 per 100,000 population.^2^ While TB incidence has decreased in the United States, remaining cases may be more challenging to treat.^3^ A key determinant is the co-prevalence of diabetes mellitus (DM), which now affects 11.4% of the US population and at least 20% of patients who fall ill from TB.^4^,^5^ DM increases the risk of developing active TB disease by three times compared to those without DM;^6^ it additionally contributes to poor treatment outcomes such as delayed microbiological clearance, development of drug resistance, relapse, and even death.^7^–^10^

One of the causes of poor treatment outcomes for persons with TB and DM (TB-DM) may be inadequate anti-TB drug exposure. Patients with TB-DM are more likely to have risk factors for delayed or reduced absorption of anti-TB medications, such as higher body weight, older age, drug–drug interactions, gastroparesis, and impaired gastric hydrochloric acid secretion.^11^–^13^ Thus, therapeutic drug monitoring (TDM) of serum anti-TB drug concentrations and personalized dosage adjustment may be valuable tools to improve treatment outcomes in TBDM patients.^14^,^15^ In Virginia, USA, a study found that TB-DM patients had low peak drug exposure, which is common among people with delayed microbiological TB clearance.^16^ Consequently, the Virginia Department of Health implemented a bundle of interventions, including screening patients with active TB disease for DM, linkage to DM care, early intervention of programmatic TDM in all patients with TB-DM, and personalized dosage adjustment as needed.^17^ With these interventions, TB-DM patients had a significantly shorter time to microbiological clearance, measured by time to sputum culture negativity, than patients with TB-DM pre-intervention.^18^ Other state TB programs have adopted a similar approach. Nevertheless, programs implementing TDM remain in the minority across the country due to the lack of comparative data and perceived cost and complexity.

The main objective of this study was to compare treatment outcomes in patients with active pulmonary TB disease and DM cared for in programs with routine TDM and those in programs that did not use TDM.

## METHODS

### Subjects

A retrospective cohort study was performed from January 2018 to December 2020 among patients diagnosed with sputum culture-positive pulmonary TB disease and DM treated at the health departments in four states in the United States (Virginia, Tennessee, Arizona, and New Mexico). State programs were selected based on known proportions of TB patients with DM and routine implementation of TDM. Florida, a state where health departments do access TDM, and New Jersey, a state without routine TDM, were initially selected but unable to provide timely data prior to the pre-selected sample size being reached. Each state health department collected data from their respective TB registry for all patients who were aged ≥18 years, with one or more sputum culture-positive samples, had documented DM, and had rifampin (RIF) susceptible pulmonary TB. Demographic and clinical data included were sex at birth, history of DM, history of other comorbidities, renal and hepatic function tests, date of first positive sputum culture for *Mycobacterium tuberculosis* complex, date of treatment initiation with conventional isoniazid (INH), RIF, ethambutol and pyrazinamide, date of negative sputum culture results after initiation of therapy, glycosylated hemoglobin A1c (HbA1c) level, chest imaging reports, anti-TB dosages, total duration of TB treatment, adverse effects, and among those with TDM performed, estimated peak drug concentrations for RIF and INH.

### Procedures

In all the US states, including the four states in this cohort, all cases of active TB disease are reported to the health department and assigned to a nurse manager and private or public health TB clinician. Directly observed therapy is administered, and sputum samples are collected per the Centers for Disease Control and Prevention guidelines with some state differences (Table 1).^3^

**Table 1:** Sputum sample collection and TDM protocols in different states

US state	TDM performed or not?	Sputum sample collection
Arizona	TDM not performed	Collect 3 consecutive sputum smear and culture at treatment initiation and then monthly until 2 consecutive sputum cultures are negative>
New Mexico	TDM not performed	Collect 3 consecutive sputum smears and culture at treatment initiation and then weekly until 3 consecutive sputum smears are negative and 2 consecutive sputum cultures are negative
Tennessee	TDM to be done after 1–2 weeks after treatment initiation for all anti-TB drugs. The dose is increased for concentrations below the expected peak range then follow-up drug testing is repeated 1–2 weeks for only those drugs that were dose adjusted	Collect 3 consecutive sputum smears and cultures weekly for 8 weeks until the sputum smear-negative and then collect 3 sputum cultures monthly until sputum culture converts to negative
Virginia	TDM to be done 2 weeks after treatment initiation for only isoniazid and rifampin. The dose is increased for concentrations below the expected peak range. No follow-up drug testing is performed unless microbiological or clinical failure	Collect 3 consecutive sputum smears and cultures at treatment initiation and then 1 sputum culture every 7–10 days for maximum 3 months, until 2 consecutive sputum cultures are negative

TDM = therapeutic drug monitoring.

For states implementing TDM, serum drug concentrations are collected for patients after daily or five times a week anti-TB treatment for at least 1–2 weeks. Patients are observed taking their medication in the morning in a fasting state or with limited food intake. Later, venous blood is collected after 2 and 6 h and sent to the Infectious Disease Pharmacokinetics Laboratory at the University of Florida, Gainesville, FL, USA, where validated high-performance liquid chromatography or gas chromatography results are reported in the expected μg/mL range. The expected peak serum concentration range for INH is 3–6 μg/mL and for RIF 8–24 μg/mL.^14^,^19^,^20^ TB consultants then interpret the TDM results and adjust dosages as needed. For example, INH dosage is increased from 300 mg to 450 mg and RIF from 600 mg to 900 mg.^17^

### Statistical analysis

We calculated a sample size of 170 to detect a significant difference for the 2-month sputum culture conversion, assuming a power of 80% and a confidence interval of 95%, and prior effect size pre- and post-intervention in Virginia.^18^ De-identified data were combined from the four sites and assigned to two groups: states that did not implement TDM, the control group (Arizona and New Mexico); and states that implemented TDM with dosage adjustments, the interventional group (Tennessee and Virginia). When appropriate, demographic and clinical data were compared between the two groups using the χ^2^ statistic or the Student *t*-test/the Mann–Whitney *U*-test. The proportion with culture conversion at 2 months was documented and compared using a binary logistic regression model with TDM as a predictor, among other risk factors for delayed culture conversion.

### Ethics

The study protocol was reviewed and approved by the institutional review board at the University of Virginia, Charlottesville, VA, USA (#23092), and secondary approvals by state health departments were obtained as needed.

## RESULTS

A total of 170 patients with sputum culture-confirmed active pulmonary TB and DM were included in the analysis. Of these, 73 (43%) patients were from states that did not implement TDM (non-TDM group), and 97 (57%) were from states that implemented TDM (TDM group). There were no significant differences in the demographics between the two groups except for ethnicity, where more people identified as Hispanic or Latino in the non-TDM group than in the TDM group (70% vs. 27%; *P* < 0.001; Table 2). In the interventional TDM group, the anti-TB dosages were adjusted in 70/97 (72%) patients. The mean serum peak concentrations for INH and RIF were not significantly different among patients from Tennessee and Virginia. Twenty-one (45%) patients in Tennessee and 21 (42%) in Virginia had both peak INH and RIF concentrations below the expected range (Table 3). Follow-up TDM at Tennessee Health Department showed increased drug levels after dosage adjustments (mean expected peak serum concentration for INH was 4.80 μg/mL ± 1.22 and for RIF 13.15 μg/mL ± 8.33).

**Table 2:** Patient demographics

Demographic	Non-TDM (*n* = 73) *n* (%)	TDM (*n* = 97) *n* (%)	*P* value
State			
Arizona	57 (78)	—	
New Mexico	16 (22)	—	
Tennessee	—	47 (49)	
Virginia	—	50 (51)	
Age, years, mean ± SD	60 ± 14	58 ± 15	0.35
Ethnicity			<0.001
Non-Hispanic or Latino	22 (30)	71 (73)	
Hispanic or Latino	51 (70)	26 (27)	
Male sex	48 (66)	71 (73)	0.31
Cavity on chest imaging	48 (66)	62 (65)	1.00
HbA1c, %, mean ± SD	9.90 ± 3.9	9.2 ± 2.5	0.33
Total patients tested, *n*	15	75	
Positive sputum smear	54 (75)	71 (73)	0.86

TDM = therapeutic drug monitoring; SD = standard deviation; HbA1c = glycosylated hemoglobin A1c.

**Table 3:** Peak serum drug concentrations in the therapeutic drug monitoring group

Serum concentration levels^*^	Tennessee (*n* = 47) mean ± SD	Virginia (*n* = 50) mean ± SD	*P* value
INH 5 times/week	3.70 ± 2.1	2.7 ± 1.9	0.11
INH daily	2.90 ± 2.2	3.10 ± 1.4	0.75
RIF 5 times/week	6.6 ± 4.1	6.90 ± 4.4	0.86
RIF daily	7.0 ± 6.3	5.72 ± 5.1	0.47
Low serum INH levels only, *n* (%)	5 (11)	9 (18)	0.32
Low serum RIF levels only, *n* (%)	6 (13)	8 (16)	0.41
Low serum INH and RIF levels, *n* (%)	21 (45)	21 (42)	0.10
Total dosages adjusted, *n* (%)	32 (68)	38 (76)	0.33

^*^ Expected peak serum concentration range for INH: 3–6 μg/mL, and for RIF: 8–24 μg/mL.

SD = standard deviation; INH = isoniazid; RIF = rifampin.

Patients in the non-TDM control group had a significantly longer time to sputum culture conversion than in the TDM group (49 ± 27 days vs. 34 ± 23 days; *P* < 0.001). Additionally, the non-TDM group had a significantly longer total treatment duration than the TDM group, (36 ±10 weeks vs. 32 ± 9 weeks; *P* = 0.04). In the non-TDM group, 51 (70%) achieved sputum culture conversion to negative by 2 months compared to 84 (87%) in the TDM group (*P* = 0.01). There was no difference in the cure, loss of follow-up, and death numbers between the two groups (Table 4). No significant adverse events were reported in the TDM group.

**Table 4:** Treatment outcomes in groups with and without TDM

Outcomes	Non-TDM (*n* = 73) mean ± SD	TDM (*n* = 97) mean ± SD	*P* value
Days to sputum culture conversion	49 ± 27	34 ± 23	<0.001
Arizona	48 ± 27	—	
New Mexico	53 ± 29	—	
Tennessee	—	31 ± 23	
Virginia	—	38 ± 22	
Total weeks of treatment duration	36 ± 10	32 ± 9	0.04
Arizona	34 ± 10	—	
New Mexico	41 ± 6	—	
Tennessee	—	33 ± 12	
Virginia	—	32 ± 8	
2-month culture conversion, *n* (%)	51 (70)	84 (87)	0.01
Arizona	43	—	
New Mexico	8	—	
Tennessee	—	42	
Virginia	—	42	
Death, *n* (%)	3 (4)	3 (3)	1.00
Cured, *n* (%)	68 (93)	92 (95)	0.51
Loss of follow-up, *n* (%)	2 (3)	2 (2)	1.00

TDM = therapeutic drug monitoring; SD = standard deviation.

We performed a binary logistic regression analysis for predictors of 2-month culture conversion as this was a programmatic benchmark, and adjusted for age, cavity on chest imaging, sex, sputum smear results at TB presentation (positive or negative), and TDM implementation. Patients with a lung cavity were less likely to achieve 2-month culture conversion (adjusted odds ratio [aOR] 0.16, 95% confidence interval [CI] 0.05–0.56, *P* = 0.004), while patients that underwent TDM were significantly more likely to achieve sputum cultures conversion at 2 months (aOR 3.05, 95% CI 1.35–6.86, *P* = 0.007). No other variable approached significance in the model.

## DISCUSSION

In this retrospective multi-state observational cohort, we found that in states that implemented TDM, patients with active pulmonary TB disease and DM were more likely to convert their sputum cultures to negative earlier and have shorter total treatment durations than patients in states that did not implement TDM. Programmatic quality metrics and determination of total treatment duration often use sputum culture conversion by 2 months, given the association between positivity at 2 months and relapse.^14^,^18^ Expectedly, those with the cavitary disease on chest imaging were significantly less likely to culture convert by 2 months, but in adjusted analyses, the use of TDM was significantly more likely to result in 2-month culture conversion.

The duration of TB treatment is usually determined using patient risk factors, clinical improvement, and time-to-culture conversion or failure to culture convert after 2 months of treatment. Patients with risk factors for slow culture conversion, such as DM or the presence of a lung cavity, are often considered for extended therapy of 9 months.^3^,^14^ Our study argues that by implementing TDM, there is a higher likelihood of early culture conversion. Therefore, the total treatment duration may not need to be extended for those with DM that access TDM and dose adjustment. As programs consider which patient populations may be most suited for even shorter course regimens, hastening the time to sputum culture conversion may be of even greater importance.^21^

Multiple studies have demonstrated that patients with suboptimal pharmacokinetics have a higher rate of treatment failure, drug resistance, and relapse.^19^,^22^ Despite international TB treatment guidelines that recommend personalized management of TB disease, data for the use of TDM in programmatic settings are limited to case series or timing as salvage where TDM is performed only after a patient has manifested a slow response to therapy.^18^,^23^ In addition, there are advantages in grouping patients by the US state of treatment, given that practice patterns are similar within states. We also intentionally grouped two states with and two states without TDM implementation to account for any between-state differences in practice. These findings are important because DM has led to delayed culture conversion and worse treatment outcomes in nearly every prior study, but here we found those states that implemented TDM achieved culture conversion as fast or faster than is typically observed for pulmonary TB patients without DM.^3^,^14^ The improvement of the 2-month microbiological outcome and shortened treatment duration for the population with DM suggests that broader implementation be considered among other groups at risk for delayed culture conversions, such as those with HIV, malabsorption, or alcohol use disorders.

Our study had some limitations, such as the retrospective design and lack of controlled randomization of the intervention. To accurately describe and measure the utility of TDM and its impact on TB treatment outcomes, prospective and randomized control trials should be conducted. However, this may be challenging for health department programs to conduct. Thus, it may be more feasible to implement TDM initially and analyze their pre- and post-TDM implementation data and decide if it is of value to that state. Nonetheless, we attempted to strengthen our design by calculating the sample size from the prior study of TDM implementation in patients with TB and DM in Virginia.^18^ We observed similar effect size of improvement in time-to-culture conversion as in the prior work. We also restricted the cohort to TB patients with DM and further prospective research is needed to study the impact of TDM in patients with TB and other comorbid diseases, such as chronic kidney disease, chronic liver disease, alcoholism, etc. Another limitation is that not all states collected sputum samples similarly (Table 1). For example, the state of Arizona collected sputum samples monthly instead of weekly; however, in comparison to New Mexico, patients from Arizona had a non-significantly shorter time to culture conversion and total treatment duration, which suggests that Arizona was not driving differences in outcomes between TDM and non-TDM states due to sparser sampling (Table 3). Also, while the TDM protocol was slightly different in Tennessee and Virginia, peak serum concentrations and proportions of dosage adjustment were similar. Finally, data remain limited regarding the specific serum therapeutic ranges for anti-TB medication; however, our findings suggest that when dosages are increased based on a minimum peak concentration, patients have improved treatment outcomes, and therefore such levels represent meaningful targets.^15^

## CONCLUSION

Our cohort highlights the impact of TDM on the microbiological outcome of sputum culture conversion. Personalized dosage adjustment was feasible in two states studied, Virginia and Tennessee, and improved the time to sputum culture conversion. Few interventions hasten time to microbiological cure, and assurance of the most rapid time-to-culture conversion may be even more critical as programs consider shorter-course regimens and which patient subtypes may be eligible.^21^ Other state TB programs currently not performing TDM should trial implementation within their own state-specific context.
